# Evolution of *ALOG* gene family suggests various roles in establishing plant architecture of *Torenia fournieri*

**DOI:** 10.1186/s12870-018-1431-1

**Published:** 2018-09-20

**Authors:** Wei Xiao, Ziqing Ye, Xinran Yao, Liang He, Yawen Lei, Da Luo, Shihao Su

**Affiliations:** 1 State Key Laboratory of Biocontrol and Guangdong Key Laboratory of Plant Resources, School of Life Sciences, Sun Yat-sen University, Guangzhou, 510275 China; 2 Institute of Transformative Bio-Molecules (WPI-ITbM), Nagoya University, Furo-cho, Chikusa-ku, Nagoya, Aichi 464-8601 Japan

**Keywords:** ALOG family, Phylogeny, Duplication, MIXTA-like, Plant architecture, *Torenia fournieri*

## Abstract

**Background:**

ALOG (Arabidopsis *LSH1* and *Oryza G1*) family with a conserved domain widely exists in plants. A handful of ALOG members have been functionally characterized, suggesting their roles as key developmental regulators. However, the evolutionary scenario of this gene family during the diversification of plant species remains largely unclear.

**Methods:**

Here, we isolated seven *ALOG* genes from *Torenia fournieri* and phylogenetically analyzed them with different *ALOG* members from representative plants in major taxonomic clades. We further examined their gene expression patterns by RT-PCR, and regarding the protein subcellular localization, we co-expressed the candidates with a nuclear marker. Finally, we explored the functional diversification of two *ALOG* members, *TfALOG1* in *euALOG1* and *TfALOG2* in *euALOG4* sub-clades by obtaining the transgenic *T. fournieri* plants.

**Results:**

The *ALOG* gene family can be divided into different lineages, indicating that extensive duplication events occurred within eudicots, grasses and bryophytes, respectively. In *T. fournieri*, seven *TfALOG* genes from four sub-clades exhibit distinct expression patterns. TfALOG1–6 YFP-fused proteins were accumulated in the nuclear region, while TfALOG7-YFP was localized both in nuclear and cytoplasm, suggesting potentially functional diversification. In the 35S:*TfALOG1* transgenic lines, normal development of petal epidermal cells was disrupted, accompanied with changes in the expression of *MIXTA*-like genes. In 35S:*TfALOG2* transgenic lines, the leaf mesophyll cells development was abnormal, favoring functional differences between the two homologous proteins. Unfortunately, we failed to observe any phenotypical changes in the *TfALOG1* knock-out mutants, which might be due to functional redundancy as the case in Arabidopsis.

**Conclusion:**

Our results unraveled the evolutionary history of *ALOG* gene family, supporting the idea that changes occurred in the *cis* regulatory and/or nonconserved coding regions of *ALOG* genes may result in new functions during the establishment of plant architecture*.*

**Electronic supplementary material:**

The online version of this article (10.1186/s12870-018-1431-1) contains supplementary material, which is available to authorized users.

## Background

Surveying the evolutionary history of plants, gene duplication plays a major role in promoting speciation [1–3]. The occurrence of duplicated genes is often related to the achievement of new functions, including those involved in plant architectures, which cause the extraordinary abundance of plant morphologies [2, 4–6].

Gene duplication generates a large proportion of genes and further forms different sizes of gene families with diverse functions, evident in *TCP* (*TEOSINTE BRANCHED 1*, *CYCLOIDEA* and *PROLIFERATING CELL NUCLEAR ANTIGEN FACTOR*), *KNOX* (*KNOTTED HOMEOBOX*) and *MADS* (*MINI CHROMOSOME MAINTENANCE 1*, *AGAMOUS*, *DEFICIENS*, and *SERUM RESPONSE FACTOR*) families [6]. *TCP* family genes encoding bHLH (basic helix-loop-helix) transcription factors are divided into three sub-clades, involved in plant developmental processes such as branching, cell size and flower shape [7–10]. KNOX family members consist in the class I KNOX proteins, which mainly regulate shoot apical meristems and leaf complexity, and the class II KNOX proteins, involved in cell wall biosynthesis [11–16]. The *MADS* gene family is another large family well-known for its functions in regulating different floral organs identities [17–20]. Although the evolutionary history and functional diversification have been well studied among some classical gene families, there are numerous families yet to be studied.

ALOG (Arabidopsis *LSH1* and *Oryza G1*) proteins are key developmental regulators among land plants, which share a highly conserved ALOG domain (also known as DOMAIN OF UNKNOWN FUNCTION 640 / DUF640) [21]. However, functional studies were only carried out in several limited species, including rice (*Oryza sativa*), *Arabidopsis thaliana*, as well as tomato (*Solanum lycopersicum*). *TAWAWA1* (*TAW1*) and *LONG STERILE LEMMA1* (*G1*) are two *ALOG* genes in rice: *TAW1* affects inflorescence architecture while *G1* represses the growth of sterile lemma in spikelet [22, 23]. In *A. thaliana*, *ORGAN BOUNDARY 1* (*OBO1*) and *OBO4*, encoding ALOG family proteins, are involved in boundary formation, which function redundantly with other genes [24, 25]. An ALOG member, TERMINATING FLOWER (TMF) in tomato, acts as a putative transcriptional regulator, affecting flower and leaf architecture [26–28]. These studies suggest functional divergence within *ALOG* gene family; however, there is still a lack of evolutionary analysis of this gene family linked to the diversified functions.

In this study, we aimed to investigate the evolutionary history of ALOG gene family, and for that, we used *T. fournieri* as material due to its importance in horticulture. Based on the phylogenetic analysis, the extensive gene duplication events were observed among three major taxonomic plant lineages: eudicots, grasses and bryophytes. In *T. fournieri*, seven *TfALOG* genes from four subclades showed distinct expression patterns and we further ectopically expressed two *ALOG* members, *TfALOG1* and *TfALOG2* from distinct sub-clades. Our results showed different functions of two ALOG proteins, favoring that changes in *cis*-regulatory and nonconserved coding regions would be important for the neo-functionalization of *ALOG* gene family.

## Methods

### Molecular cloning

Plant DNA was extracted by 2% CTAB from juvenile leaves and total RNA was extracted from different tissues, using Plant RNA Kit (Omega Bio-Tek, Guangzhou, China) according to the manufacturer’s instructions. The first strand cDNA were synthesized using PrimeScript RT reagent Kit with gDNA Eraser (Takara, Beijing, China). To isolate *ALOG* and *MIXTA*-like genes in *T. fournieri*, protein sequences of LSHs (ALOG members from *A. thaliana*) and AmMIXTAs (MIXTA-like factors from *Antirrhinum majus*) were used to blast in the in-house transcriptome of *T. fournieri*. The corresponding cDNA sequences were obtained and their relative ORFs (Open Reading Frames) were predicted through an online program ORF-Finder (https://www.ncbi.nlm.nih.gov/orffinder/). After further blasting in Basic Local Alignment Search Tool (https://blast.ncbi.nlm.nih.gov/Blast.cgi), the candidate genes were maintained. To verify the sequences from the transcriptome, gene-specific primers were designed for PCR, and genomic DNA and cDNA were used as templates. The PCR products were cloned into pMD19-T vector (Takara, Beijing, China) prior to sequencing. Sequences were then analyzed in MEGA7 to identify the gene structures [29]. Primers used in this study were listed in Additional file 1: Table S1.

### Phylogeny analysis and motif-based sequence analysis

ALOG genes from *T. fournieri* were cloned in this study. To figure out the evolutionary history of *ALOG* genes in plants, we collected homologous gene sequences from Phytozome version 12 and other databases (https://phytozome.jgi.doe.gov). The reference database versions from Phytozome were as follows: *Physcomitrella patens*: version 3.3; *Sphagnum fallax*: version 0.5; *Oryza sativa*: version 7.0; *Zea mays*: version PH207 v1.1; *Sorghum bicolor*: version 3.1.1; *Brachypodium distachyom*: version Bd21–3 v1.1; *Aquilegia coerulea*: version 3.0; *Arabidopsis thaliana*: version TAIR10; *Populus trichocarpa*: version 3.0; *Daucus carota*: version 2.0; *Mimulus guttatus*: version 2.0; *Solanum lycopersicum*: version iTAG2.4.). Other databases of two Lamiales species including *Sesamum indicum* and *Utricularia gibba* were also referred [30, 31].

Alignments of nucleotide and amino acid sequences were performed in MEGA7 [29]. Using MRBAYES v.3.2.1 with the GTR + I + G nucleotide substitution model, the aligned sequences were analyzed with 300,000,000 generations and a 1000 generation sample frequency [32]. *ALOG* gene sequences of the representative plant species were submitted to motif-based sequence analysis website (MEME; http://meme-suite.org/tools/meme) for motif mining under the parameters: -nmotifs 50, −minw 6, −maxw 50.

### RT-PCR and quantitative RT-PCR (qRT-PCR)

The templates used in the RT-PCR were synthesized by the RevertAid First Strand cDNA Synthesis Kit (Thermo Fisher Scientific, Shanghai, China). Seven cloned *ALOG* genes were examined. *β-actin* (*TfACT3*) was the internal control of the RT-PCR as previously reported [33]. For qRT-PCR, we obtained the cDNA under the protocol of PrimeScript RT reagent Kit with gDNA Eraser (Takara, Beijing, China). Using LightCycler 480 Real-Time PCR System (Roche, Shanghai, China), qRT-PCR was performed based on the manual. *β-actin* (*TfACT3*) was used as an internal control of the qRT-PCR. Three biological replicates were performed to calculate the SD in qRT-PCR assays. Graphs were produced with GraphPad Prism 5. Primers used in this study were listed in Additional file 1: Table S1.

### Protein signal prediction and subcellular localization

The nuclear localization signals were predicted in http://mleg.cse.sc.edu/seqNLS/. The open reading frame (ORF) of each *T. fournieri ALOG* gene was fused with a yellow fluorescent protein (YFP) in the C-terminal and was inserted into pA7 plasmid (provided by Prof. Hongwei Xue from Institute of Plant Physiology and Ecology, Chinese Academy of Science). The leaf mesophyll protoplasts of *Arabidopsis thaliana* were extracted from two-week plants and followed by PEG-induced transformation, as previously described [34]. All the ALOG-YFP plasmids were co-transformed with a nuclear marker, ARF19IV-mCherry, as previously used [35]. The YFP and mCherry signals were observed using a confocal laser scanning microscopy Zeiss7 DUO NLO.

### Plant growth and transformation of *T. fournieri*

Plants were kept under 16-h-light and 8-h-dark greenhouse with 70–80% relative humidity at 22–24 °C. Sterile seedlings were obtained as previously reported [33]. The binary vector, pCAMBIA1302, was used for transformation. The ORFs of two target genes *TfALOG1* and *TfALOG2* were driven by 35S promoter. The construction of *TfALOG1*-Cas9 vector followed the protocol provided by Prof. Yaoguang Liu from South China Agricultural University [36]. *Agrobacterium tumefaciens* strain EHA105 was used for plant transformation as previously described [37]. Transgenic plants were screened on the 1/2 MS media containing hygromycin and were identified by PCR.

### Scanning electron microscopy (SEM)

Mature petals of *T. fournieri* were dissected and taped on slide glasses. Multiple layers of impression materials were used to generate the epoxy replicas of petals as previously described in [38]. The epoxy replicas were sputtered with gold particles before observing under a scanning electron microscope Jeol JSM 6360LV (Jeol, Tokyo, Japan). The images were adjusted by Adobe PHOTOSHOP CS6 (Adobe, San Jose, CA, USA).

## Results

### Isolation of *ALOG* genes from *T. fournieri*

We used *T. fournieri* as a representative model of Asterids lineage eudicot, from which we isolated seven *ALOG* members that we named as *TfALOG1*–7 (Additional file 1: Figure S1A). Generally, the *ALOG* genes from *T. fournieri* encode small proteins with about 200 amino acids. Only two members out of seven, namely *TfALOG3* and *TfALOG7,* have an intron with 374 bp and 255 bp, respectively (Additional file 1: Figure S1A). Multiple alignment indicated these ALOG proteins contain one conserved ALOG domain, with highly variable N-/C- terminals (Additional file 1: Figure S1B).

### *ALOG* gene family has undergone multiple times of independent duplication and/or loss events during the diversification of land plants

**Fig. 1:**
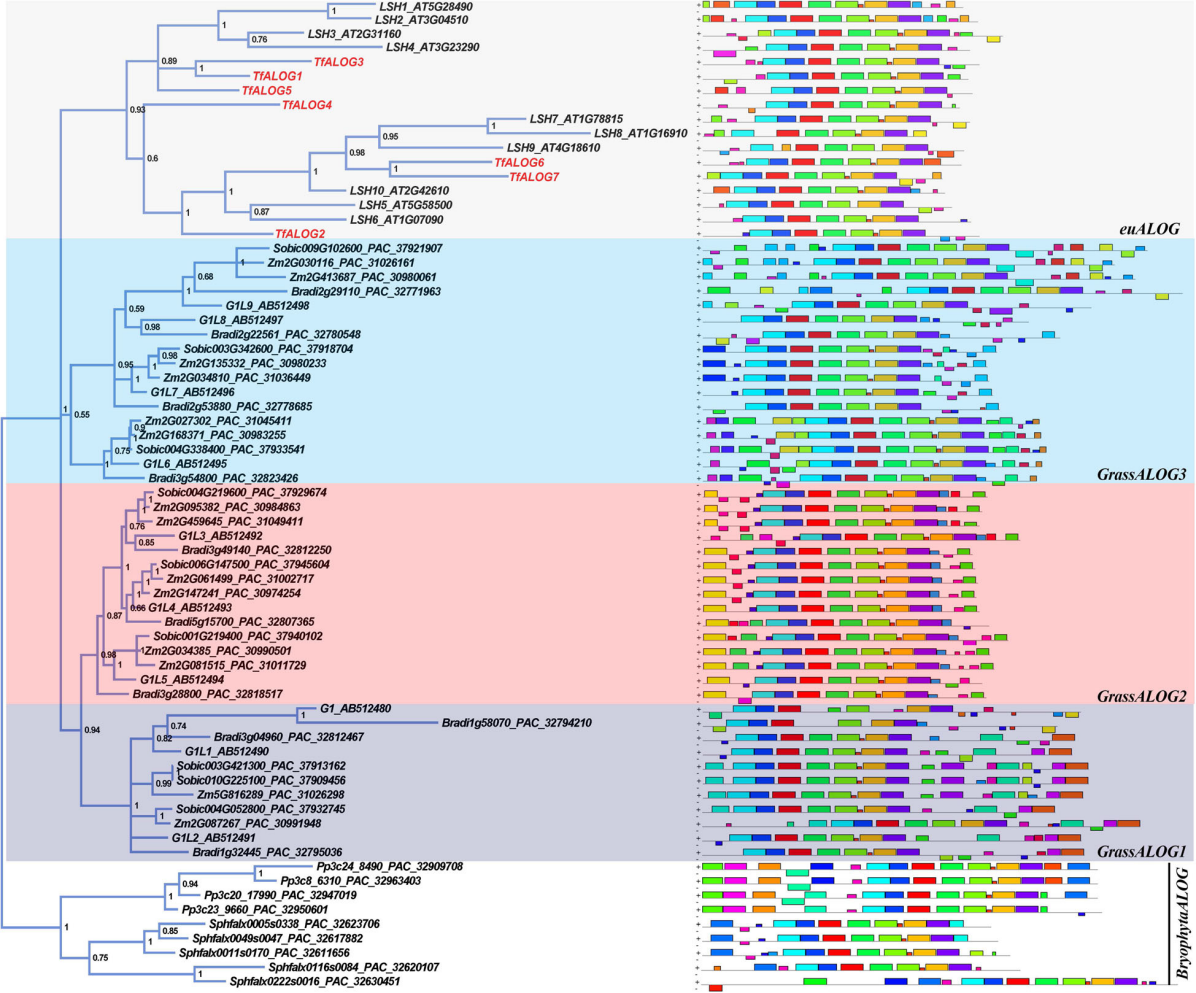
Bayesian phylogram (left) and motif analysis (right) of *ALOG* genes in three plants lineages. *Physcomitrella patens* and *Sphagnum fallax* were selected as outer groups. The Bayesian posterior probability is located in each node and the accession number can be found in each sequence. Seven *ALOG* genes cloned from *Torenia fournieri* were presented in red. The motif diagrams were generated in MEME and different colors represent different motifs

To reconstruct the evolutionary history of *ALOG* gene family, we in-depth mined the *ALOG* members using BLAST (Basic Local Alignment Search Tool) in different plants genomes. *ALOG* genes are present in the basal land plants like *Physcomitrella patens* and *Sphagnum fallax* (Fig. 1). There are 4 and 5 *ALOG* members characterized from *P. patens* and *S. fallax* genome, respectively (Fig. 1). Members from one species clustered together, indicating that these duplication events happened independently after speciation (Fig. 1). Due to the differences in their sequences, we designated the *PpALOG*s and *SfALOG*s as out-groups in later phylogeny studies.

In angiosperms, *ALOG* genes from monocots and eudicots clustered independently (Fig. 1). The grass *ALOG* genes from *Zea mays*, *Oryza sativa*, *Sorghum bicolor* and *Brachypodium distachyon*, were divided into three clades named *GrassALOG1/2/3* (Fig. 1). The *GrassALOG3* clade was further divided into three sub-clades because of its motif differences (Fig. 1). The phylogenetic tree of grass *ALOG*s also provided us two indications: first, *ALOG* genes have been duplicated in their common ancestors; second, independent duplication and/or loss events occurred together with the divergence of Poaceae (Fig. 1). There has been a whole genome duplication event in *Z. mays* after its divergence from *S. bicolor* [39]. However, only *GrassALOG2* and *GrassALOG3* clades members supported the duplication event, as there were two *ZmALOG* paralogues corresponding to one *SbALOG* gene in these two clades (Fig. 1).

**Fig. 2:**
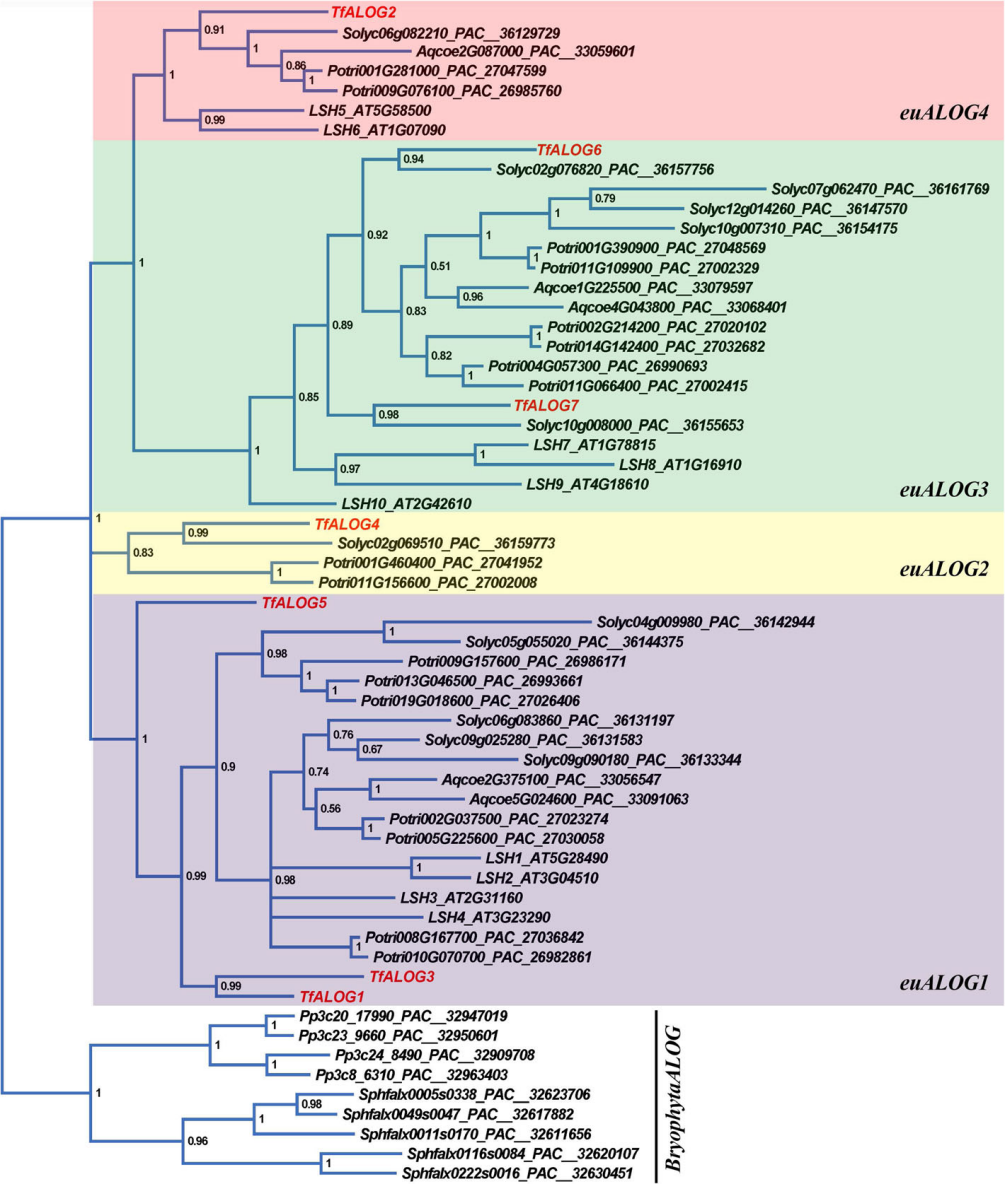
Bayesian phylogram of *ALOG* genes in eudicots. *Physcomitrella patens* and *Sphagnum fallax* were chosen as outer groups. The Bayesian posterior probability is located in each node and the accession number can be found in each sequence. Seven *ALOG* genes cloned from *Torenia fournieri* were presented in red

We further focused on eudicots, and selected five representative species for phylogeny analysis: *Aquilegia coerulea*, a basal eudicots; *A. thaliana* and *Populus trichocarpa*, two Rosids lineage species; *S. lycopersicum* and *T. fournieri*, two Asterids lineage species. The phylogenetic tree of the *ALOG* genes from eudicots separated into four clades, designated as *euALOG1*, *euALOG2*, *euALOG3* and *euALOG4* (Fig. 2). These four clades of *euALOG*s were also evident when more Asterids *ALOG* genes (from *Daucus carota*, *Mimulus guttatus*, *S. lycopersicum*, *Sesamum indicum*, *Utricularia gibba* and *T. fournieri*) were added to the phylogenetic tree (Additional file 1: Figure S2). Apart from the ancestral duplication events before the divergence of eudicots, frequent duplication and/or loss of *euALOG* members also occurred along with speciation (Fig. 2). Since different clades of *euALOG*s have been duplicated independently, we inferred that the *ALOG* gene family might play various roles in different biological processes.

### Expressional analysis of *ALOG* genes in *T. fournieri*

**Fig. 3:**
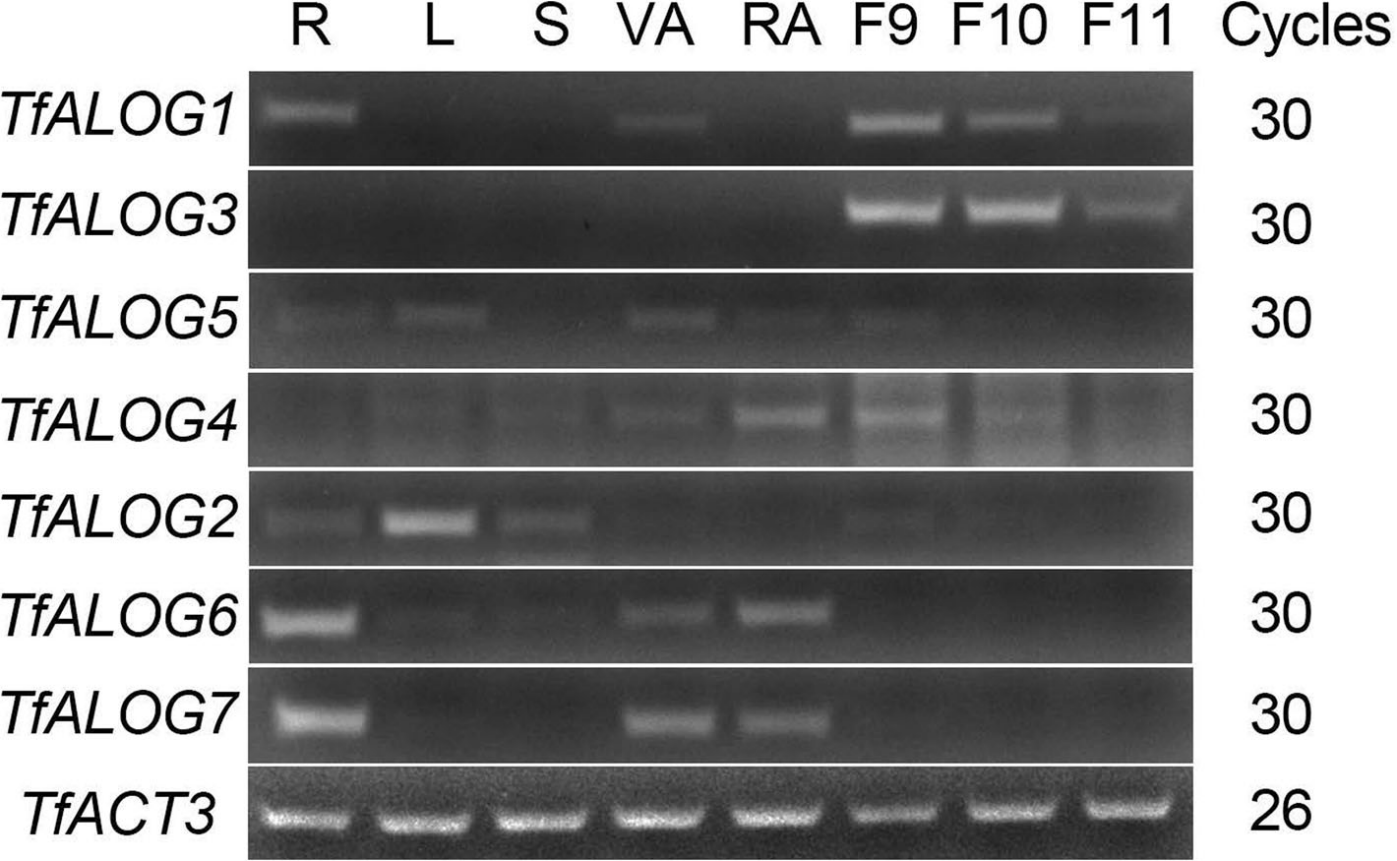
Reverse transcription polymerase chain reaction analysis (RT-PCR) of *TfALOG* genes. Gene names are shown on the left sides; PCR cycles are shown on the right sides in each gene. β-actin (*TfACT3*) was used as an internal control. R, root; S, stem; L, leaf; VA, vegetative apexes; RA, reproductive apexes; F9, stage 9 flower bud; F10, stage 10 flower bud; F11, stage 11 flower bud

Since the expression pattern of one gene is always closed related to its function, we examined the expression of 7 *ALOG* genes from *T. fournieri* by performing RT-PCR in different plant tissues. The phylogenetic tree indicated that *TfALOG1* and *TfALOG3* clustered closely, together with *TfALOG5*, belonging to the *euALOG1* clade; *TfALOG6* and *TfALOG7* clustered in the *euALOG3* clade; while *TfALOG2* and *TfALOG4* were found in *euALOG2* and *euALOG4* clades, respectively (Fig. 2). The expression patterns of *ALOG* genes were highly correlated with their phylogeny, except for *TfALOG*5, which showed a different expression pattern compared with its paralogues (Fig. 3). *TfALOG1* and *TfALOG*3 were highly expressed in different flower buds, while *TfALOG*5 was expressed in roots, leaves and vegetative and reproductive apexes; *TfALOG*6 and *TfALOG*7 were highly expressed in roots and vegetative and reproductive apexes; *TfALOG*2 was highly accumulated in leaf and *TfALOG*4 was found almost in all tissues we examined (Fig. 3). The distinct expression patterns of *T. fournieri ALOG* genes implied that this family might have generated various functions in the establishment of plant architecture.

### Subcellular localization of TfALOG-YFP fused proteins

**Fig. 4:**
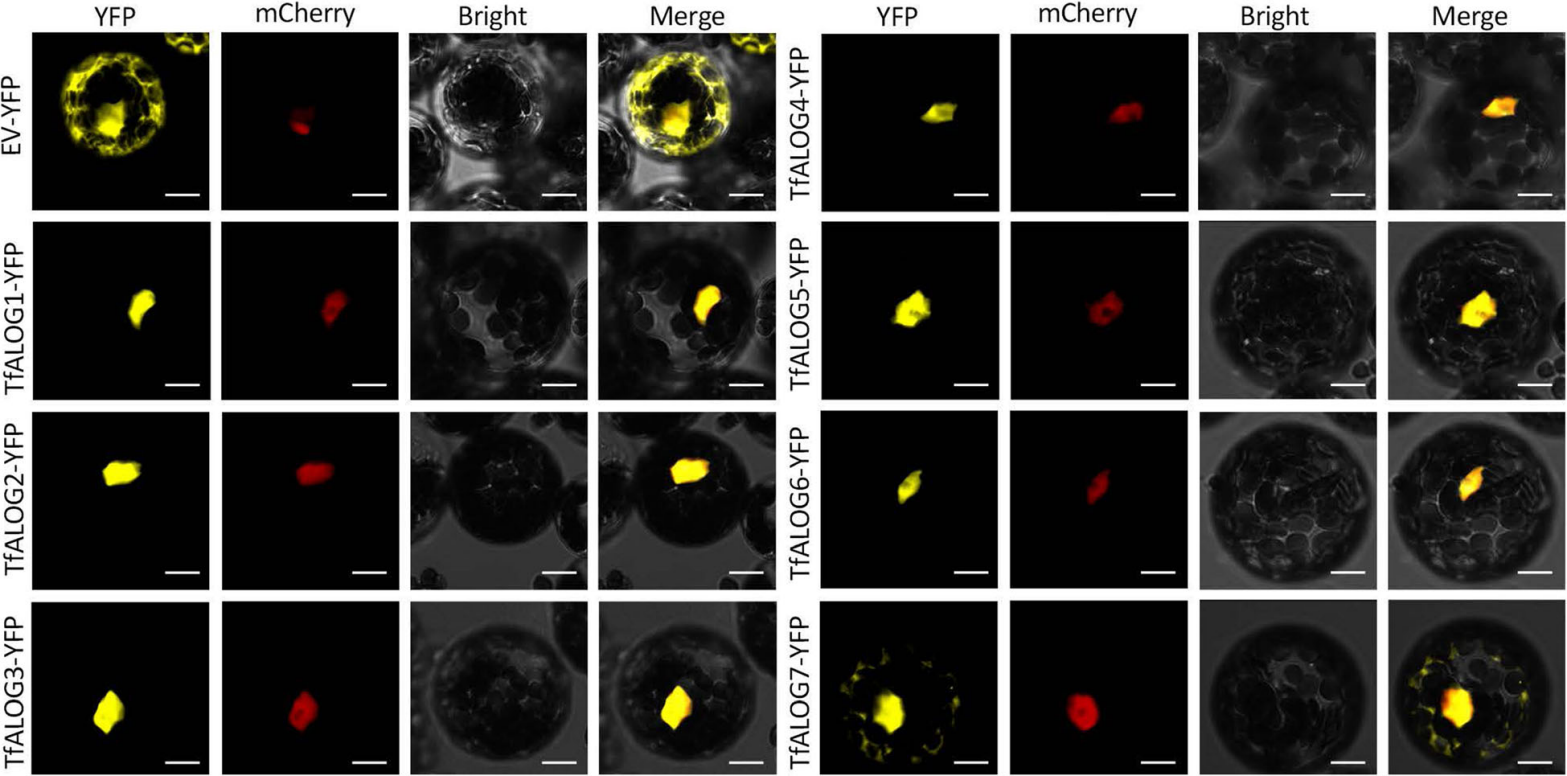
Subcellular localization of TfALOG-YFP fused proteins. Signals from YFP, mCherry, bright field and merged channels are shown in each assay; EV-YFP is an empty vector as a control; nuclear marker ARF19IV-mCherry plasmids were co-transformed with different YFP constructs; scale bars: 10 μm

The subcellular localization of a protein is linked to its function, thus we analyzed the potential nuclear localization signals within the seven TfALOG proteins using online tools. The results showed that all the TfALOG proteins had nuclear localization signals in their N-terminals (Additional file 1: Figure S3). To verify the predicted results, we fused the open reading frame of each *TfALOG* gene with a yellow florescence protein (YFP) tag and co-transformed into Arabidopsis mesophyll protoplasts with a nuclear marker ARF19IV-mCherry. The empty plasmid with an YFP was used as a control, which existed in both nuclear and cytoplasm regions (Fig. 4). TfALOG1–6 YFP-fused proteins were all co-localized with the nuclear marker, indicating their accumulation in the nuclear region (Fig. 4). One exception was from TfALOG7 YFP-fused protein, which was localized both in nuclear and cytoplasm, suggesting potentially functional diversification (Fig. 4). These results supported that TfALOGs might basically work as transcriptional factors / cofactors, as previously reported.

### Phenotypic analysis of 35S:*TfALOG1* and 35S:*TfALOG2* transgenic plants

**Fig. 5:**
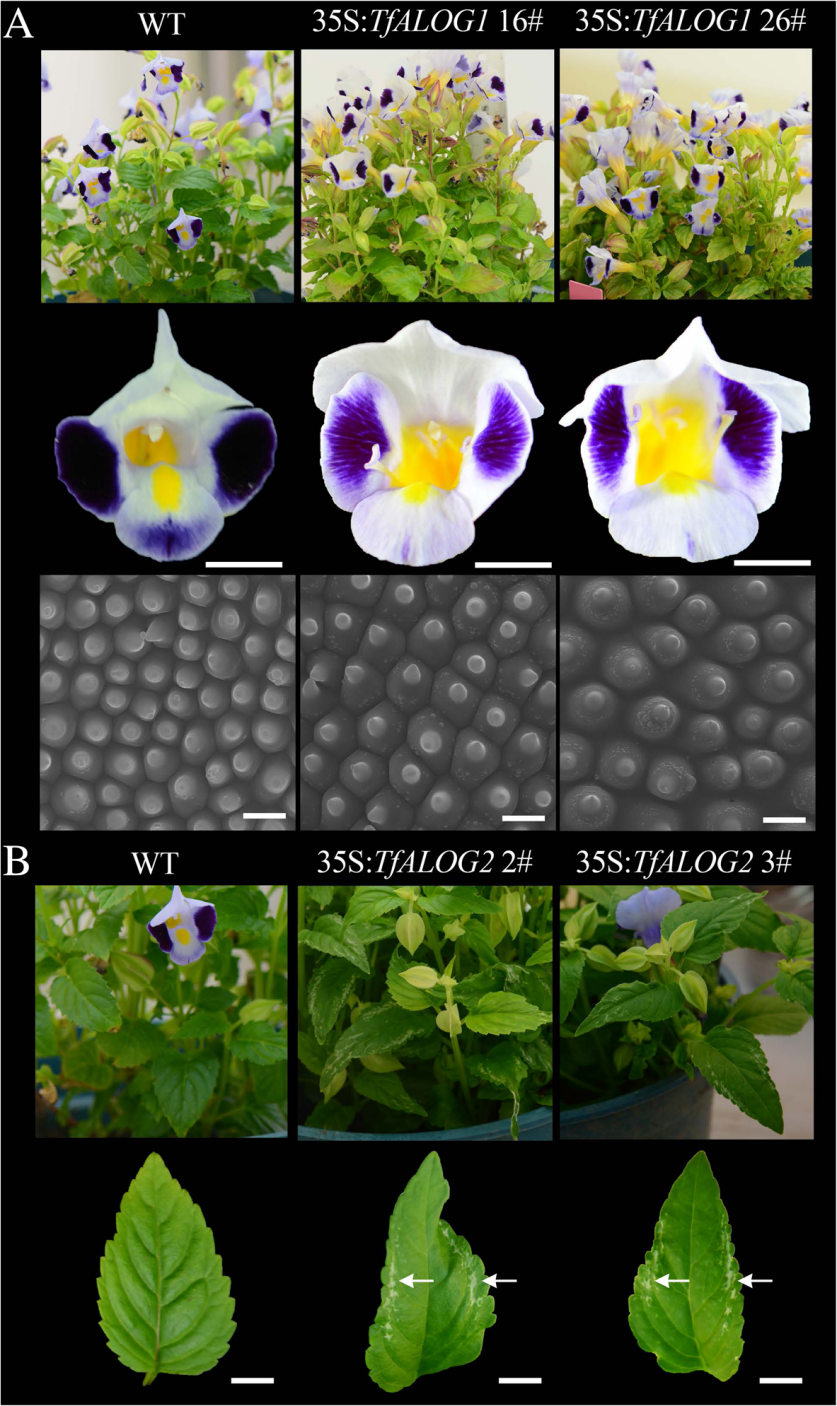
Phenotypic analysis of wild type (WT), 35S:*TfALOG1* and 35S:*TfALOG2* transgenic plants. **a** Three rows represent the over-all plants, flowers (bars: 10 mm) and epidermal cells in petal lobes (bars: 20 μm), two independent lines 35S:*TfALOG1* 16# and 35S:*TfALOG1* 26# were used for analysis. **b** Two rows represent the over-all plants and leaves (bars: 10 mm), two independent lines, 35S:*TfALOG2* 2# and 35S:*TfALOG2* 3# were used for analysis; white arrows indicate developmental defects on leaves

To explore the functions of *TfALOG* genes, we ectopically expressed *TfALOG1* from *euALOG1* clade and *TfALOG*2 from *euALOG4* clade in *T. fournieri*. In the 35S:*TfALOG1* transgenic plants (14 lines showed stable phenotypes out of 30 transgenic lines), the pigmentation and shape of flowers became abnormal compared with the wild type (Fig. 5 a). The light-violet petal background color was less intensely than WT (Fig. 5 a). We further observed the epidermal cells of petals by scanning electronic microscopy, and found that the conical cells in the lobe regions became flat together with an increase in cell size (Fig. 5 a). Furthermore, we observed other phenotypes in 35S:*TfALOG1* transgenic lines, which included changes in petal shape, elongated yellow region in petal tube, as well as changed leaf color (Additional file 1: Figure S4A). These results demonstrated *TfALOG1* as an important player during plant organ development. However, we failed to observe any phenotypical change in the *TfALOG1* knock-out mutants (among 10 genetically edited lines), which may be due to functional redundancy among this family, consistent with previous studies from Arabidopsis (Additional file 1: Figure S6) [24, 25, 40].

When we ectopically expressed *TfALOG*2 (10 lines showed stable phenotypes out of 19 transgenic lines), normal leaf development was affected, which was consistent with the high expression of *TfALOG*2 in leaves (Fig. 5b). The mesophyll cells of marginal leaf regions failed to form in the 35S:*TfALOG2* transgenic plants and this was always linked with changes in leaf shape, suggesting its function in leaf development (Fig. 5b). We did not observe any difference in the flowers of 35S:*TfALOG2* transgenic plants (Additional file 1: Figure S4B).

**Fig. 6:**
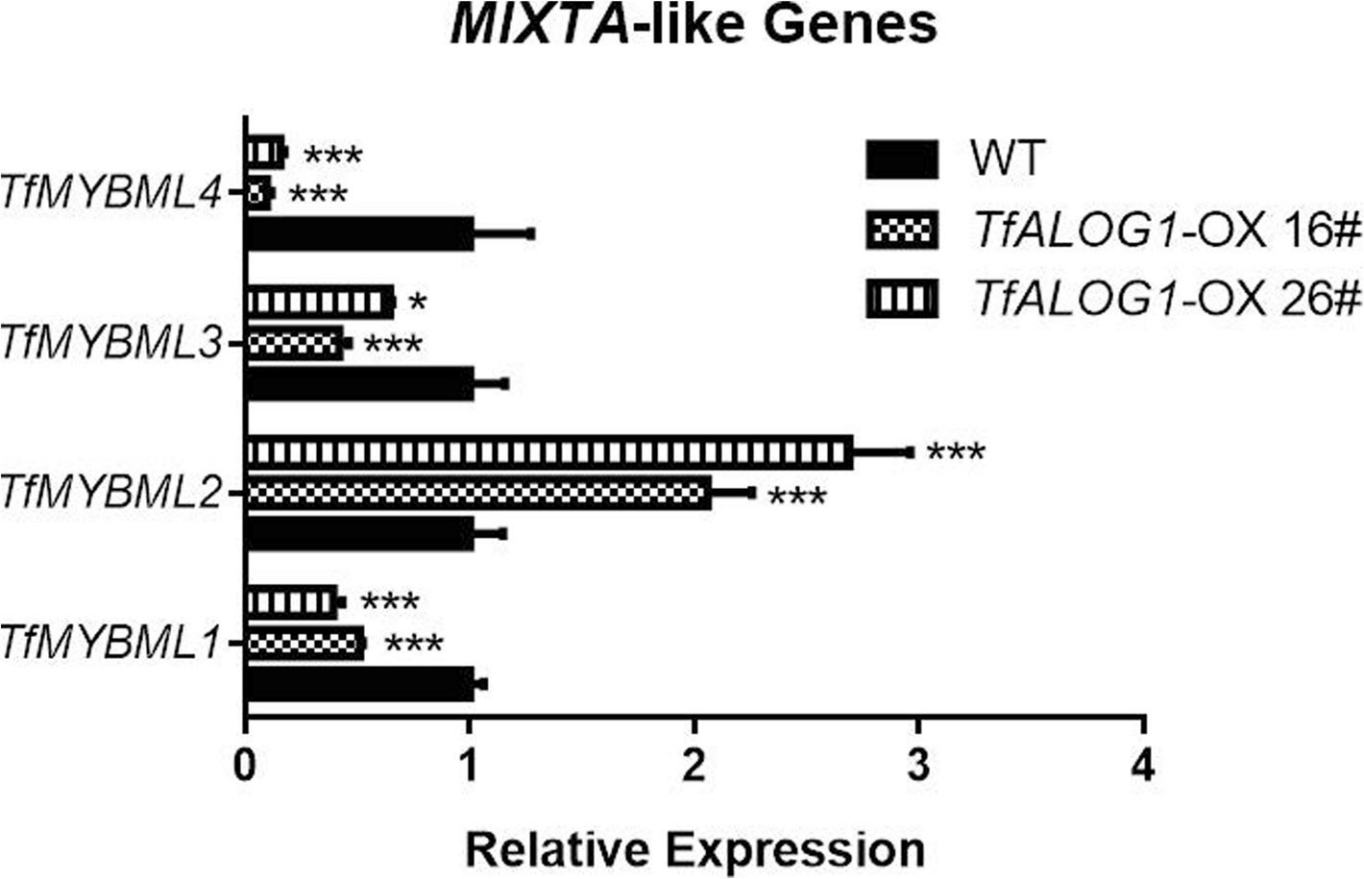
Relative expression of *TfMIXTA* genes in wild type (WT) and 35S:*TfALOG1* transgenic plants. Relative expression of *TfMIXTA* genes in different plants was determined by qRT-PCR in the stage 10 flower buds. Error bars represent ±1 SD from three biological replicates; statistically significant differences were marked by asterisks with *: *P* < 0.05. ***: *P* < 0.001

It has been reported that MIXTA-like MYB transcription factors were responsible for the differentiation of conical epidermal cells [41–43], thus we cloned four *MIXTA*-like factors from *T. fournieri* (Additional file 1: Figure S1C). All four *MIXTA*-like genes were highly expressed in the petal lobe regions, consistent with the existence of conical epidermal cells (Additional file 1: Fig. S5). We also found that the three flower-expressed *TfALOG1/3/4* genes were preferentially expressed in the petal tube regions, which differed from the expression of *MIXTA*-like genes (Additional file 1: Figure S5). Hence, we inferred antagonistic roles between *TfALOG*s and *TfMIXTA*s. To test this hypothesis, we checked the expression of *TfMIXTA*s in the 35S:*TfALOG1* transgenic lines. Three out of four *MIXTA*-like genes, *TfMIXTA1*, *TfMIXTA3* and *TfMIXTA4*, were significantly down-regulated in the 35S:*TfALOG1* transgenic flowers, while *TfMIXTA2* was up-regulated in the transgenic plants (Fig. 6). These indicated functional differences among the *MIXTA*-like genes [41, 44–46]. These data threw lights on how *TfALOG1* was involved in the regulation of flower development.

## Discussion

### Evolutionary scenario of *ALOG* gene family

The ALOG domain was originated from the XerC/D-clade of tyrosine recombinases containing a novel class of DIRS-1 transposon, which belongs to the tyrosine recombinase/phage integrase N-terminal DNA-binding domain superfamily [21]. We found that the *ALOG* genes evolved from the basal land plants and independently duplicated multiple times during the diversification of plant species, which was consistent with a previously study [40].

The phylogenetic analysis showed that, *ALOG* family members from different large plant lineages could be easily distinguished (Figs. 1 and 2). For example, the grass *ALOG* members were distinct from *ALOG*s in either eudicots or bryophytes. We did not detect any ALOG member from published genomes of the green algae lineage (chlorophytes) indicating that ancient *ALOG* gene emerged after the generation of land plants. Although multiple *ALOG* members are found from the basal land plant genomes, they have never been functionally studied. Phylogenetic analysis suggested that these multiple bryophyta *ALOG* paralogues share a common ancestor with distinct motifs in the N-terminal. In grasses, there has been a significant increase in the number of *ALOG* members, companying with the increased complexity in plant architecture. We were surprised by the phylogenetic tree in which all the grass ALOG members were separated from the eudicots, suggesting a possible functional divergence (Fig. 1). In eudicots, the *ALOG* family members were further divided into four clades, although independent loss of genes occurred in different eudicots species (Fig. 2). The general four *euALOG* clades were also supported by the Asterids *ALOG* phylogenetic tree, which indicated functional conservation within different clades of *euALOG* genes (Additional file 1: Figure S2).

### Functions of ALOG family members

The predicted secondary structure of an ALOG domain shares four conserved helices with a zinc ribbon inserted between the helices two and three [21]. This secondary structure is likely to confer a DNA binding activity to the ALOG domain, possibly making the protein function as a transcriptional factor [21].

Until now, there are only three rice ALOG members that have been functionally characterized: G1 from grassALOG1 clade, TAW1/G1 L5 from grassALOG3 clade and G1 L6/TH1/BLS1/BSG1/AFD1 from grassALOG3 clade [22, 23, 47–51]. The expression patterns and functions of different grass *ALOG* gene clades are divergent: The mRNA of *G1* is found in sterile lemma as well as in the basal region of palea, and is involved in the repression of lemma identity during the sterile lemma development [22]; *TAW1* is expressed in various meristems including shoot apical, axillary, inflorescence and branch meristems; gain-of-function *taw1-D* mutants result in an increase of inflorescence branches [23]; *TH1* transcripts are ubiquitous in multiple tissues, and the *th1* mutant plants show defects both in vegetative and reproductive organ development [47, 50]. Transcriptional activity assays suggest that G1 and TAW1 likely act as transcriptional activators, while TH1 works as a transcriptional repressor [22, 23, 51]. These results indicate that grass *ALOG* genes functions evolved after gene duplication events.

In the eudicots, to our knowledge, only five ALOG members have been functionally addressed: LSH1, LSH3 and LSH4 from Arabidopsis, TMF from tomato and LjALOG1 from *Lotus japonicus* [24–27, 40, 52]. Over-expression of Arabidopsis *ALOG* genes leads to changes in seedling development and boundary formation; however, no obvious phenotype could be detected in either *LSH1* antisense transgenic lines or *lsh3 lsh4* double mutants, indicating functional redundancy in Arabidopsis [24, 25, 40]. From our phylogenetic tree, there were four euALOG1 clade members from Arabidopsis, which might function redundantly (Fig. 1). Although multiple closed paralogues exist in tomato, the single mutant of *TMF* shows strong phenotypes with early flowering time and conversion from an inflorescence to single abnormal flower [26]. The TMF protein can physically interact with multiple transcription factors such as BOP-like proteins, supporting the hypothesis of TMF working as a transcriptional cofactor that controls inflorescence architecture [26, 27]. In *Lotus japonicus*, an ALOG gene family member *LjALOG1*, highly expressed in nodule, has a novel role in controlling nodulation of legume species [52].

### Functional divergence in *T. fournieri ALOG* family

In this study, we isolated seven *ALOG* genes from *T. fournieri* and noticed that the N/C-terminals of these ALOG proteins were rather divergent from each other even among those from the same clade (Fig. 1). In order to detect their functional divergence, we ectopically expressed two *TfALOG* members from different clades. Ectopic expression of *TfALOG2* led to abnormal leaf development, while in 35S:*TfALOG1* transgenic lines, normal flower development was affected (Fig. 5). These results indicated that different functions have been evolved from changes in the variable regions among these ALOG proteins.

### TfALOG1 may be involved in petal differentiation

*MIXTA*-like *MYB* genes play various roles in plant epidermis including trichome development in *Gossypium hirsutum*, cuticle formation in Arabidopsis and *T. fournieri*, and epidermal cell differentiation in *Thalictrum* [42, 53, 54]. As an important pollination syndrome, different studies conclude that conical cells on petal epidermis are also determined by *MIXTA*-like *MYB* genes [41–46].

In the 35S:*TfALOG1* transgenic lines, conical epidermal cells in the lobe regions became flat with large amount of granules adhered onto their surface (Fig. 5 a). We also found multiple *MIXTA*-like genes down-regulated in the 35S:*TfALOG1* transgenic lines, supporting that *TfALOG1* may regulate petal epidermis differentiation by directly or indirectly inhibiting the expression of *MIXTA*-like genes in the petal tube region (Fig. 6).

## Conclusions

Our study unveiled the evolutionary scenario of *ALOG* family in land plants. We observed that multiple gene duplication events independently occurred in different plant linages. We also systematically studied seven *ALOG* genes from an Asterids species, *T. fournieri*, and found functional differences of TfALOG proteins in the establishment of plant architecture. In the future, it is of interest to explore how *TfALOG1* is involved in petal epidermal cell differentiation and how *TfALOG2* is recruited to leaf development. Our work offers new insights of functional divergence among plant *ALOG* gene family.

## Additional file

Additional file 1: **Figure S1.** Structures of *TfALOG*s and *TfMIXTA*s. (A) Gene structures of *TfALOG*s. (B) Multiple alignment of TfALOG proteins. The red line indicates the conserved DUF 640 domain. (C) Gene structures of *TfMIXTA*s. Box represents exon region and line represents intron region. **Figure S2.** Bayesian phylogram of *ALOG* genes in Asterids. *Physcomitrella patens* and *Sphagnum fallax* were chosen as outgroups. The Bayesian posterior probability is located in each node and the accession number can be found in each sequence. Seven *ALOG* genes cloned from *Torenia fournieri* were presented in red. **Figure S3.** The predicted NLSs (Nuclear Localization Signal) of TfALOG proteins. The NLSs were highlighted in red. **Figure S4.** Other phenotypes of transgenic plants. (A) Petal and leaf phenotypes analysis of wild type (WT) and 35S:*TfALOG1* transgenic plants; two independent lines 35S:*TfALOG1* 16# and 35S:*TfALOG1* 26# were used for analysis. Two dorsal, two laterals and one ventral petal were dissected from the flower. (B) Flower phenotypes of 35S:*TfALOG2* transgenic plants; two independent lines 35S:*TfALOG2* 2# and 35S:*TfALOG2* 3# were used for analysis. **Figure S5.** Relative expression of *TfMIXTA* and *TfALOG* genes. Relative expression of *TfMIXTA* and *TfALOG* genes in different tissues of wild type flowers were determined using qRT-PCR. WT10-L, petal lobes from stage 10 flowers; WT10-T, petal tubes from stage 10 flowers; WT11-L, petal lobes from stage 11 flowers; WT11-T, petal tubes from stage 11 flowers; WT12-L, petal lobes from stage 12 flowers; WT12-T, petal tubes from stage 12 flowers. Error bars represent ±1 SD from three biological replicates. **Figure S6.** Details of transgenic plants. (A) Relative expression of *TfALOG1* in WT and two independent lines 35S:*TfALOG1* 16# and 35S:*TfALOG1* 26#. Error bars represent ±1 SD from three biological replicates. (B) Relative expression of *TfALOG2* in WT and two independent lines 35S:*TfALOG2* 2# and 35S:*TfALOG1* 3#. Error bars represent ±1 SD from three biological replicates. (C) Designed CRISPR/Cas9 targets. Targets were highlighted in yellow, start and end codons were marked in red. (D) Sequence alignments of *TfALOG1* in two TfALOG1 knock-out lines, designed targets were marked by red boxes. **Table S1.** Primers used in this study. (DOCX 5469 kb)

## Abbreviations

ALOG: Arabidopsis LSH1 and Oryza G1
DUF640: DOMAIN OF UNKNOWN FUNCTION 640
G1: LONG STERILE LEMMA1
KNOX: KNOTTED HOMEOBOX
MADS: MINI CHROMOSOME MAINTENANCE 1, AGAMOUS, DEFICIENS, and SERUM RESPONSE FACTOR
OBO: Organ Boundary
ORF: Open reading frame
SEM: Scanning electron microscopy
TAW1: TAWAWA1
TCP: TEOSINTE BRANCHED 1, CYCLOIDEA and PROLIFERATING CELL NUCLEAR ANTIGEN FACTOR
TMF: TERMINATING FLOWER
YFP: Yellow fluorescent protein
