# Supplementary material for: Evolution of ALOG gene family suggests various roles in establishing plant architecture of Torenia fournieri
Source: BMC Plant Biol. 2018 Sep 20;18:204. doi: 10.1186/s12870-018-1431-1 (PMC6148777; doi:10.1186/s12870-018-1431-1)
Supplement: Supplementary file 1 — Figure S1. Structures of TfALOGs and TfMIXTAs. (A) Gene structures of TfALOGs. (B) Multiple alignment of TfALOG proteins. The red line indicates the conserved DUF 640 domain. (C) Gene structures of TfMIXTAs. Box represents exon region and line represents intron region. Figure S2. Bayesian phylogram of ALOG genes in Asterids. Physcomitrella patens and Sphagnum fallax were chosen as outgroups. The Bayesian posterior probability is located in each node and the accession number can be found in each sequence. Seven ALOG genes cloned from Torenia fournieri were presented in red. Figure S3. The predicted NLSs (Nuclear Localization Signal) of TfALOG proteins. The NLSs were highlighted in red. Figure S4. Other phenotypes of transgenic plants. (A) Petal and leaf phenotypes analysis of wild type (WT) and 35S:TfALOG1 transgenic plants; two independent lines 35S:TfALOG1 16# and 35S:TfALOG1 26# were used for analysis. Two dorsal, two laterals and one ventral petal were dissected from the flower. (B) Flower phenotypes of 35S:TfALOG2 transgenic plants; two independent lines 35S:TfALOG2 2# and 35S:TfALOG2 3# were used for analysis. Figure S5. Relative expression of TfMIXTA and TfALOG genes. Relative expression of TfMIXTA and TfALOG genes in different tissues of wild type flowers were determined using qRT-PCR. WT10-L, petal lobes from stage 10 flowers; WT10-T, petal tubes from stage 10 flowers; WT11-L, petal lobes from stage 11 flowers; WT11-T, petal tubes from stage 11 flowers; WT12-L, petal lobes from stage 12 flowers; WT12-T, petal tubes from stage 12 flowers. Error bars represent ±1 SD from three biological replicates. Figure S6. Details of transgenic plants. (A) Relative expression of TfALOG1 in WT and two independent lines 35S:TfALOG1 16# and 35S:TfALOG1 26#. Error bars represent ±1 SD from three biological replicates. (B) Relative expression of TfALOG2 in WT and two independent lines 35S:TfALOG2 2# and 35S:TfALOG1 3#. Error bars represent ±1 SD from three biological replicates [file 12870_2018_1431_MOESM1_ESM.docx]

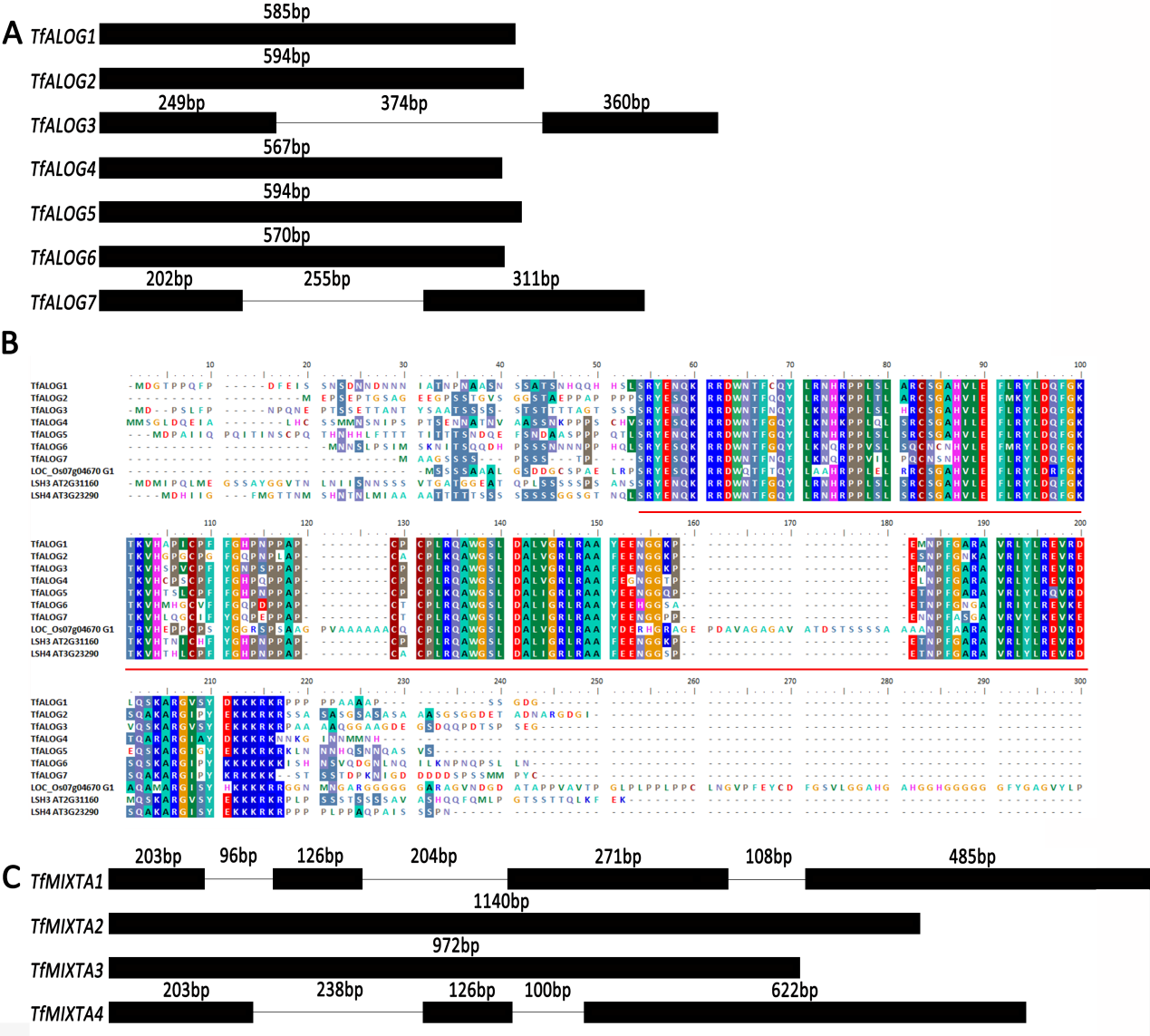


Fig. S1. Structures of *TfALOG*s and *TfMIXTA*s. (A) Gene structures of *TfALOG*s. (B) Multiple alignment of TfALOG proteins. The red line indicates the conserved DUF 640 domain. (C) Gene structures of *TfMIXTA*s. Box represents exon region and line represents intron region.

Fig. S2. Bayesian phylogram of *ALOG* genes in Asterids. *Physcomitrella patens* and *Sphagnum fallax* were chosen as outgroups. The Bayesian posterior probability is located in each node and the accession number can be found in each sequence. Seven *ALOG* genes cloned from *Torenia fournieri* were presented in red.


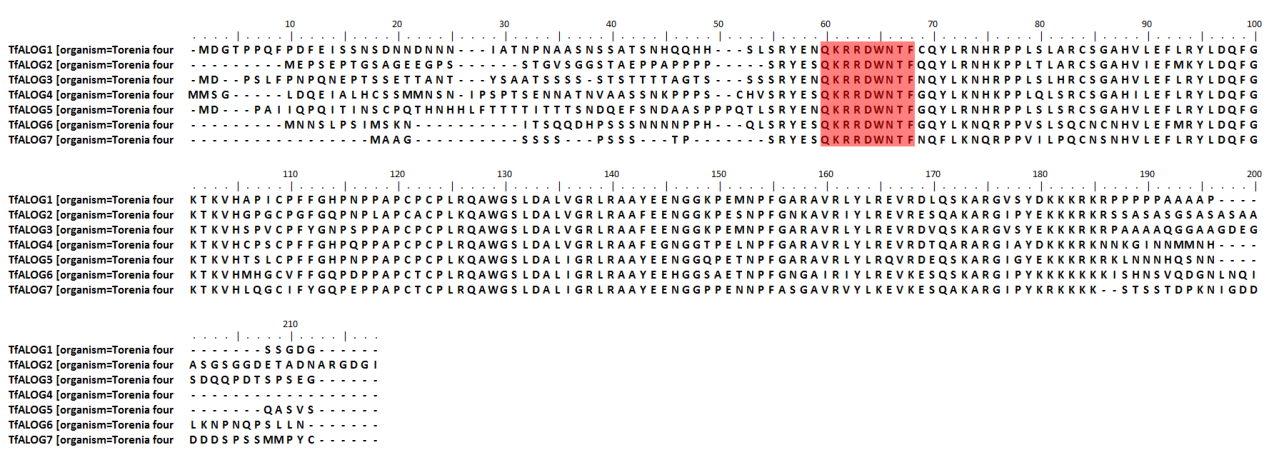


Fig. S3. The predicted NLSs (Nuclear Localization Signal) of TfALOG proteins. The NLSs were highlighted in red.


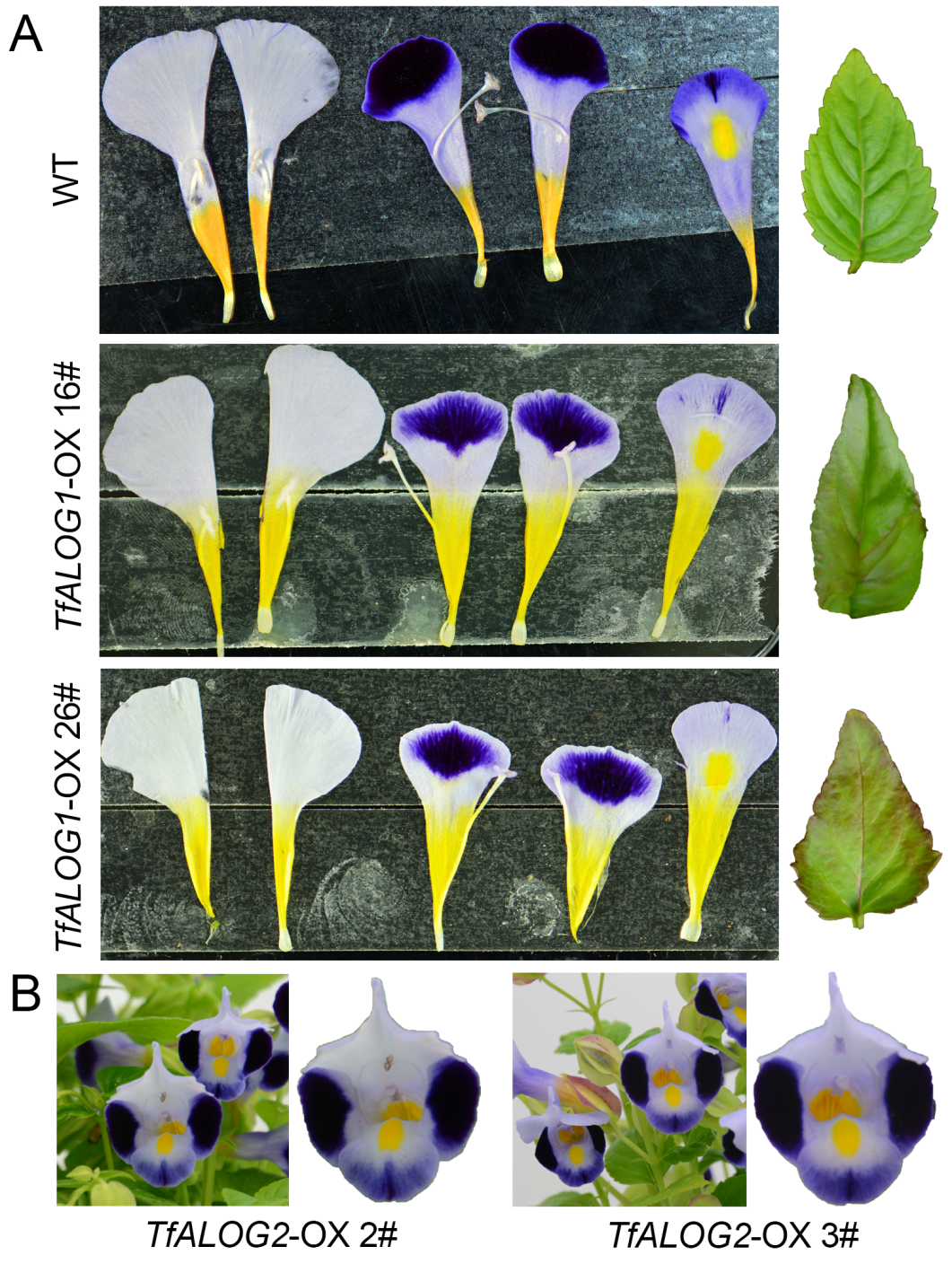


Fig. S4 Other phenotypes of transgenic plants. (A) Petal and leaf phenotypes analysis of wild type (WT) and 35S:*TfALOG1* transgenic plants; two independent lines 35S:*TfALOG1* 16# and 35S:*TfALOG1* 26# were used for analysis. Two dorsal, two laterals and one ventral petal were dissected from the flower. (B) Flower phenotypes of 35S:*TfALOG2* transgenic plants; two independent lines 35S:*TfALOG2* 2# and 35S:*TfALOG2* 3# were used for analysis.

Fig. S5. Relative expression of *TfMIXTA* and *TfALOG* genes. Relative expression of *TfMIXTA* and *TfALOG* genes in different tissues of wild type flowers were determined using qRT-PCR. WT10-L, petal lobes from stage 10 flowers; WT10-T, petal tubes from stage 10 flowers; WT11-L, petal lobes from stage 11 flowers; WT11-T, petal tubes from stage 11 flowers; WT12-L, petal lobes from stage 12 flowers; WT12-T, petal tubes from stage 12 flowers. Error bars represent ±1 SD from three biological replicates.


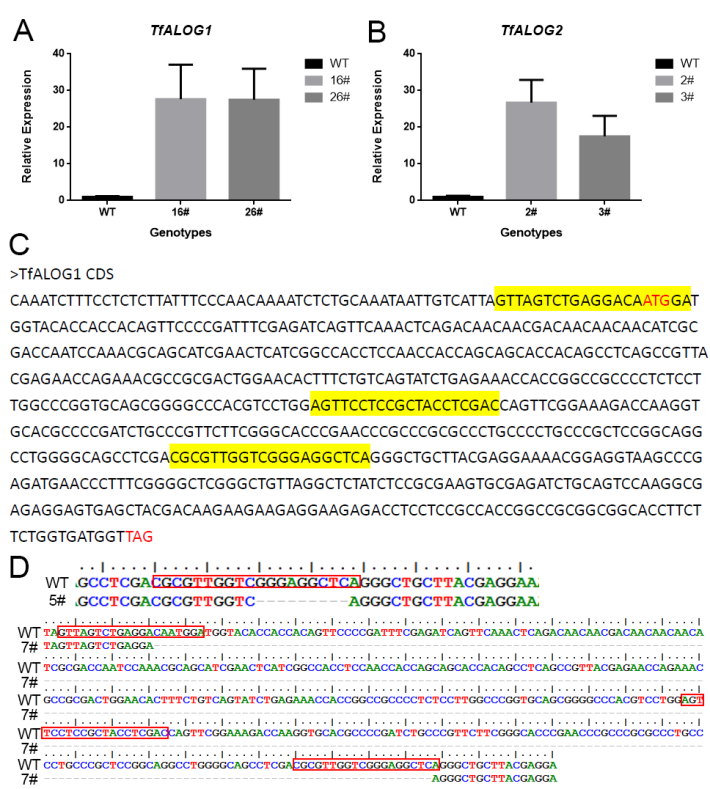


Fig. S6. Details of transgenic plants. (A) Relative expression of *TfALOG1* in WT and two independent lines 35S:*TfALOG1* 16# and 35S:*TfALOG1* 26#. Error bars represent ±1 SD from three biological replicates. (B) Relative expression of *TfALOG2* in WT and two independent lines 35S:*TfALOG2* 2# and 35S:*TfALOG1* 3#. Error bars represent ±1 SD from three biological replicates. (C) Designed CRISPR/Cas9 targets. Targets were highlighted in yellow, start and end codons were marked in red. (D) Sequence alignments of *TfALOG1* in two TfALOG1 knock-out lines, designed targets were marked by red boxes.

Table S1 Primers used in this study

| Primers | Sequences(5'-3') | Proposes |
| --- | --- | --- |
| TfALOG1-F | ATGGATGGTACACCACCACAG | molecular cloning |
| TfALOG1-R | ACCATCACCAGAAGAAGGTGC | molecular cloning |
| TfALOG2-F | ATGGAACCATCCGAACCAAC | molecular cloning |
| TfALOG2-R | CAAACCATCCCCACAGG | molecular cloning |
| TfALOG3-F | ATGGACCCATCATTGTTCCC | molecular cloning |
| TfALOG3-R | CCCTTCACTTGGAGACGTATC | molecular cloning |
| TfALOG4-F | ATGATGTCTGGTCTTGATCAAGAA | molecular cloning |
| TfALOG4-R | ATGATTCATCATATTATTTATGCCC | molecular cloning |
| TfALOG5-F | ATGGATCCAGCAATAATTCAACC | molecular cloning |
| TfALOG5-R | ACTAACTGACGCTTGATTATTGCTC | molecular cloning |
| TfALOG6-F | ATGAATAACTCTCTACCTTCAATCA | molecular cloning |
| TfALOG6-R | ATTTAACAAACTGGGCTGGT | molecular cloning |
| TfALOG7-F | ATGGCCGCAGGATCTTC | molecular cloning |
| TfALOG7-R | ACAATATGGCATCATAGAAGATG | molecular cloning |
| TfMYBML1-F | ATGGGAAGAGCACCATGTTG | molecular cloning |
| TfMYBML1-R | AAACTCCGGCGAATGATG | molecular cloning |
| TfMYBML2-F | ATGGGTCGATCACCATGCTGTG | molecular cloning |
| TfMYBML2-R | AAATTCCGGCGAGCGAGAA | molecular cloning |
| TfMYBML3-F | ATGGGACGAGCACCATG | molecular cloning |
| TfMYBML3-R | GCCAAACGTCTTAAGAAATCC | molecular cloning |
| TfMYBML4-F | ATGGCGAAAACGAAGGAG | molecular cloning |
| TfMYBML4-R | ACAACTGTAATTAATTACCGAGTCTTC | molecular cloning |
| TfALOG1-F | AACTCAGACAACAACGACAACAACA | RT-PCR |
| TfALOG1-R | GTAGCTCACTCCTCTCGCCTTG | RT-PCR |
| TfALOG2-F | ATGGAACCATCCGAACCAACTG | RT-PCR |
| TfALOG2-R | TCCGACCAGAGCATCCAAGC | RT-PCR |
| TfALOG3-F | GAGACCACCGCCAACACCTA | RT-PCR |
| TfALOG3-R | CGCTTCCTCTTCTTCTTCTCGTAG | RT-PCR |
| TfALOG4-F | ATGATGTCTGGTCTTGATCAAGAAA | RT-PCR |
| TfALOG4-R | CAACGGGCAGGGGCAGGGAG | RT-PCR |
| TfALOG5-F | ATGGATCCAGCAATAATTCAACC | RT-PCR |
| TfALOG5-R | GAAGAAGGGGCACAGGTTGG | RT-PCR |
| TfALOG6-F | CTACCTTCAATCATGTCCAAGAACA | RT-PCR |
| TfALOG6-R | AAGCCGACCGATCAGAGCAT | RT-PCR |
| TfALOG7-F | ATGGCCGCAGGATCTTCAT | RT-PCR |
| TfALOG7-R | TCCGTTCTCCTCATAAGCCG | RT-PCR |
| TfACT3-F | GCTGTTCTCTCCCTTTATGC | RT-PCR |
| TfACT3-R | GCACACAGAGAATAGCAAAC | RT-PCR |
| TfALOG1-qPCR-F1 | CCCCGATTTCGAGATCAGTTC | qRT-PCR |
| TfALOG1-qPCR-R1 | AGTTCGATGCTGCGTTTGG | qRT-PCR |
| TfALOG2-qPCR-F1 | AGAGTCAAGCAAAGGCGAGAG | qRT-PCR |
| TfALOG2-qPCR-R1 | AGCAGAAGCAGAAGCAGAACC | qRT-PCR |
| TfALOG3-qPCR-F1 | AATGGAGGAAAACCAGAGATGAAC | qRT-PCR |
| TfALOG3-qPCR-R1 | GCTCTGCACGTCACGAACTTC | qRT-PCR |
| TfALOG4-qPCR-F1 | GCAGGTACGAGTCCCAGAAAC | qRT-PCR |
| TfALOG4-qPCR-R1 | CTGAGGGTGTCCGAAGAAAGG | qRT-PCR |
| TfALOG5-qPCR-F1 | CTCTTCACCACCACCACCATC | qRT-PCR |
| TfALOG5-qPCR-R1 | TGTTCCAGTCTCGGCGTTTC | qRT-PCR |
| TfALOG6-qPCR-F1 | CGGTGCGATAAGGATTTACTTGAG | qRT-PCR |
| TfALOG6-qPCR-R1 | ACTGGGCTGGTTTGGATTCTTC | qRT-PCR |
| TfALOG7-qPCR-F1 | ACCCGAGAATAACCCGTTTGC | qRT-PCR |
| TfALOG7-qPCR-R1 | GTCACCATCATCGTCATCATCATC | qRT-PCR |
| TfACT3-F1 | AGCGTGAAATCGTTCGAGACA | qRT-PCR |
| TfACT3-R1 | CTTAGCTGTTTCGAGCTCTTGCT | qRT-PCR |
| TfMYBML1-F1 | TCGTCTTTCCTGTCGTCTTCTTC | qRT-PCR |
| TfMYBML1-R1 | TCTTCTCCGTGATGGTTCTGTTC | qRT-PCR |
| TfMYBML2-F1 | CGGCGGAGATGAGATGGAAC | qRT-PCR |
| TfMYBML2-R1 | GTGTCGTAGTCAGTAATCAGCAAC | qRT-PCR |
| TfMYBML3-F1 | CCTGCCATCATCATCCTCGTC | qRT-PCR |
| TfMYBML3-R1 | GTTGTTGCTGGAGTTGTCGTG | qRT-PCR |
| TfMYBML4-F1 | ACTGGTCATCTTCATCGTCATCG | qRT-PCR |
| TfMYBML4-R1 | TTTCGGGTTGTGGGTTAGCG | qRT-PCR |
| TfALOG1-F-YFP | CCAGTCGACATGGATGGTACACCACCACAG | subcellular localization |
| TfALOG1-R-YFP | CCAACTAGTACCATCACCAGAAGAAGGTGC | subcellular localization |
| TfALOG2-F-YFP | CCAGTCGACATGGAACCATCCGAACCAAC | subcellular localization |
| TfALOG2-R-YFP | CCAACTAGTCAAACCATCCCCACAGG | subcellular localization |
| TfALOG3-F-YFP | CCAGTCGACATGGACCCATCATTGTTCCC | subcellular localization |
| TfALOG3-R-YFP | CCAACTAGTCCCTTCACTTGGAGACGTATC | subcellular localization |
| TfALOG4-F-YFP | CCAGTCGACATGATGTCTGGTCTTGATCAAGAA | subcellular localization |
| TfALOG4-R-YFP | CCAACTAGTATGATTCATCATATTATTTATGCCC | subcellular localization |
| TfALOG5-F-YFP | CCAGTCGACATGGATCCAGCAATAATTCAACC | subcellular localization |
| TfALOG5-R-YFP | CCAACTAGTACTAACTGACGCTTGATTATTGCTC | subcellular localization |
| TfALOG6-F-YFP | CCAGTCGACATGAATAACTCTCTACCTTCAATCA | subcellular localization |
| TfALOG6-R-YFP | CCAACTAGTATTTAACAAACTGGGCTGGT | subcellular localization |
| TfALOG7-F-YFP | CCAGTCGACATGGCCGCAGGATCTTC | subcellular localization |
| TfALOG7-R-YFP | CCAACTAGTACAATATGGCATCATAGAAGATG | subcellular localization |
| TfALOG1-target1-F | GGTCGTTAGTCTGAGGACAATGGA | CRISPR/Cas9 construction |
| TfALOG1-target1-R | AAACTCCATTGTCCTCAGACTAAC | CRISPR/Cas9 construction |
| TfALOG1-target2-F | ATTGCCCCGATCTGCCCGTTCTT | CRISPR/Cas9 construction |
| TfALOG1-target2-R | AAACAAGAACGGGCAGATCGGGG | CRISPR/Cas9 construction |
| TfALOG1-target3-F | ATTGCGCGTTGGTCGGGAGGCTCA | CRISPR/Cas9 construction |
| TfALOG1-target3-R | AAACTGAGCCTCCCGACCAACGCG | CRISPR/Cas9 construction |
| 1302-TfALOG1-F | CATGCCATGGATGGTACACCACCACAG | Over-expression |
| 1302-TfALOG1-R | GACTAGTACCATCACCAGAAGAAGGTGC | Over-expression |
| 1302-TfALOG2-F | CATGCCATGGAACCATCCGAACCAACTG | Over-expression |
| 1302-TfALOG2-R | GACTAGTCAAACCATCCCCACAGG | Over-expression |
